# Ectopic miR-975 induces CTP synthase directed cell proliferation and differentiation in *Drosophila melanogaster*

**DOI:** 10.1038/s41598-019-42369-6

**Published:** 2019-04-15

**Authors:** Wai Kan Woo, Najat Dzaki, Shallinie Thangadurai, Ghows Azzam

**Affiliations:** School of Biological Sciences, Universiti Sains Malaysia, 11800 Penang, Malaysia

## Abstract

CTP synthase (CTPSyn) is an essential metabolic enzyme, synthesizing precursors required for nucleotides and phospholipids production. Previous studies have also shown that CTPSyn is elevated in various cancers. In many organisms, CTPSyn compartmentalizes into filaments called cytoophidia. In *Drosophila melanogaster*, only its isoform C (CTPSynIsoC) forms cytoophidia. In the fruit fly’s testis, cytoophidia are normally seen in the transit amplification regions close to its apical tip, where the stem-cell niche is located, and development is at its most rapid. Here, we report that *CTPSynIsoC* overexpression causes the lengthening of cytoophidia throughout the entirety of the testicular body. A bulging apical tip is found in approximately 34% of males overexpressing *CTPSynIsoC*. Immunostaining shows that this bulged phenotype is most likely due to increased numbers of both germline cells and spermatocytes. Through a microRNA (miRNA) overexpression screen, we found that ectopic miR-975 concurrently increases both the expression levels of CTPSyn and the length of its cytoophidia. The bulging testes phenotype was also recovered at a penetration of approximately 20%. However, qPCR assays reveal that *CTPSynIsoC* and miR-975 overexpression each provokes a differential response in expression of a number of cancer-related genes, indicating that the shared *CTPSyn* upregulation seen in either case is likely the cause of observed testicular overgrowth. This study presents the first instance of consequences of miRNA-asserted regulation upon *CTPSyn* in *D*. *melanogaster*, and further reaffirms the enzyme’s close ties to germline cells overgrowth.

## Introduction

One of the many hindrances to the discovery of a common cure for cancer is in the disease’s inherent complexity. Arguably every intersection of every pathway vital for fundamental cellular processes could, if disturbed, contribute to a cell’s transformation from a normal to cancerous entity^1^. Pathways leading to the production of nucleotides are therefore tightly regulated, as their products are not only critical for duplication and subsequent division of an emerging cancer cell^2^, but also form an integral part of energy storage^3^ and the phospholipid- producing bio-machinery^4^.

In pyrimidine synthesis, an example of an essential metabolic enzyme is CTP Synthase (CTPSyn). The enzyme catalyses the conversion of UTP to CTP via well-characterized *de novo* and salvage pathways^5,6^. Its uniqueness lies in its tendency to form filaments called cytoophidia^7^; such structures have been reported in all manners of organisms, ranging from the simple bacterium to advanced mammals^8–11^. Despite their kingdom-transcending conservation, an understanding of the exact function of CTPSyn-cytoophidia as well as the factors governing their formation remains poorly established.

Certain properties of the CTPSyn-cytoophidia within *Drosophila melanogaster* has nonetheless been revealed. For example, only isoform C of the protein (CTPSynIsoC) is actually involved in the structure’s formation^12^. Several studies have shown a correlative relationship between CTPSyn cellular concentration to the length of cytoophidia^12,13^. Under conditions whereby it is excessively available, the structure tends to become elongated^9^, whereas the opposite is true if its gene is knocked-out or mutated. This dynamism exhibited by the cytoophidium in response to surrounding changes means that it could, at the very least, serve as an indicator for normal levels of cellular nucleotide production, especially in tissues where it could be found consistently and in relatively high numbers. The drosophilid testis is one such tissue. Both macro and micro-cytoophidia are observable in primary spermatocytes^10^. Unfortunately, the distribution patterns of cytoophidia at points of spermatogenesis preceding and succeeding this stage are insufficiently characterized. The structure has not been reported in the germline or cyst stem cells surrounding the apical hub cells, nor the gonialblasts giving rise to spermatocytes. Whether cytoophidia is retained throughout ensuing meiotic events is yet another uncertainty.

A careful record of the trend of cytoophidia formation within testis will thus inadvertently provide information regarding CTPSyn levels at a specific point of the tissue’s development. Thorough characterization of its gene’s expression patterns is of interest as increased levels of the protein has been linked to several mammalian cancer types such as sarcoma^14^, hepatoma^15,16^ and leukaemia^17^. Cytoophidia-forming properties of CTPSyn is also notably often associated with tumorigenetic genes. The popular oncogene *Myc* encourages cytoophidia formation, but requires CTPSyn to exert its cellular overgrowth effects^18^. Under dysregulation, *Ack1* is linked to a poor prognosis in prostate cancer^19^; CTPSyn not only colocalizes in cytoophidia with its protein, but mutants lack total CTP content, indicating that CTPSyn function is directly responsive to Ack1^20^. IMPDH, instrumental in conferring chemo-resistance to human osteosarcoma when excessively expressed, is yet another cytoophidium-forming metabolic enzyme with co-localization as well as gene-gene interaction links to CTPSyn^21^. A relationship between CTPSyn and the exacerbation of cancers is in fact observed frequently enough that it has been recognized as a feasible target in therapy. Multiple drugs have been developed with the intention of attenuating the enzyme adequately enough to combat cancer progression^22–24^.

Here we report that when the enzyme is present in excess, it contributes to an increase in the girth of the *Drosophila* testis at its apical tip. This phenotype, herewith referred to as ‘bulging’, always coincides with detection of grossly elongated cytoophidia, a hallmark of *CTPSynIsoC* overexpression in *D*. *melanogaster*. More detailed immunostaining reveals that bulging may be attributed to larger population sizes of germline cells and spermatocytes when compared to controls. We also show that the overexpression of a singular microRNA i.e. miR-975 leads to similar testicular bulging phenotypes. Though it is shown that excessive growth may also be caused by increased cell numbers, and RT-qPCR data confirms the consequential upregulation of *CTPSynIsoC* in testes tissue overexpressing miR-975, the lengthening of cytoophidia is not observed. Our study therefore shows that whilst bulge-induction under the overexpression of *CTPSynIsoC* and miR-975 are both related to higher levels of the CTPSynIsoC enzyme, pathways effected in either case differ greatly, inferring that *CTPSynIsoC* may be involved in more mechanisms of cellular growth control than previously thought.

## Results

### Testes of flies overexpressing *CTPSynIsoC* show elongated cytoophidia and significantly greater incidence of apical testis bulging

**Figure 1:**
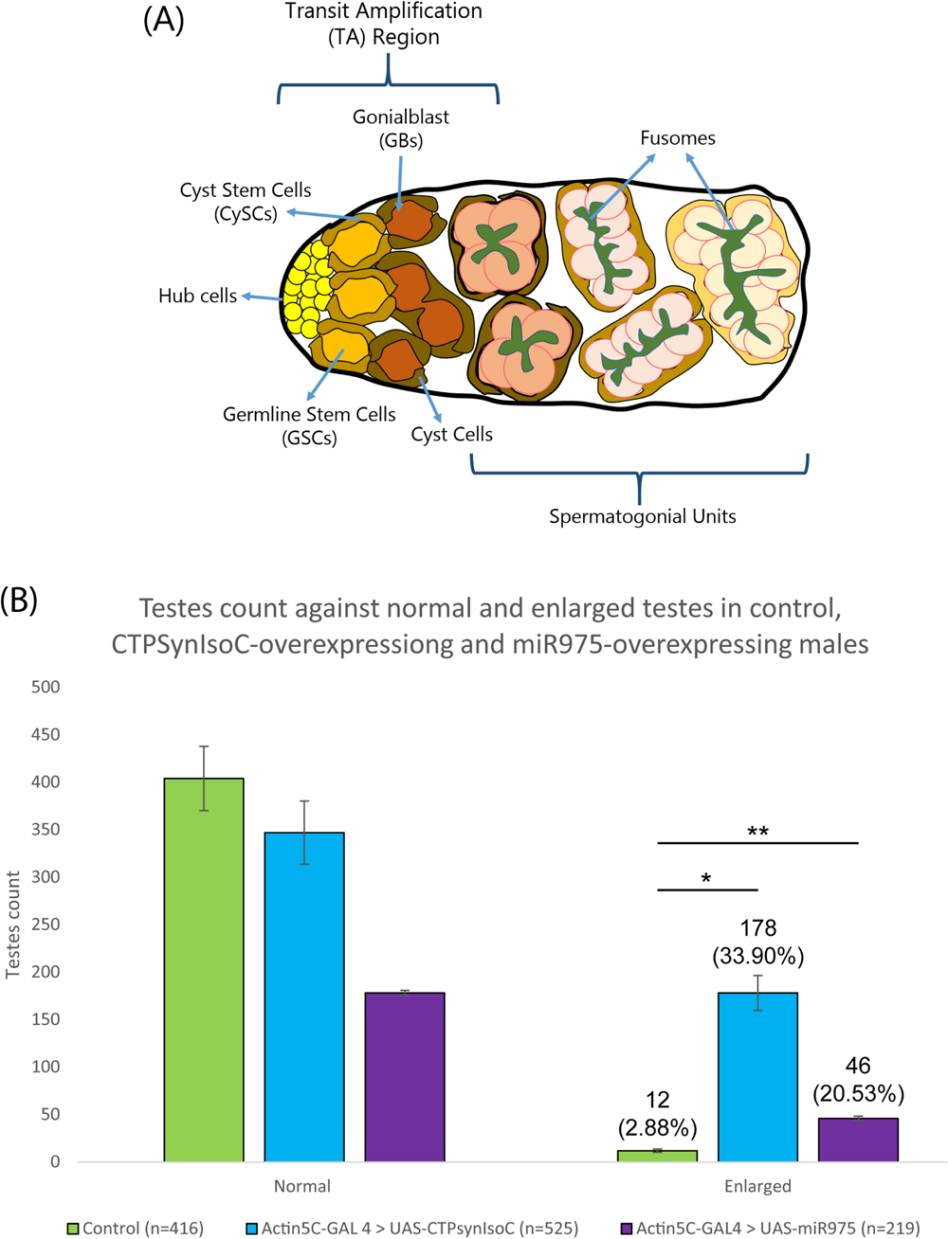
Overexpression of either *CTPSynIsoC* or miR-975 causes the bulging of testicular apical tips. (**A**) Schematic diagram of an apical tip of a testis and spermatogenesis. (**B**) Bar chart showing the numbers of phenotypically normal and enlarged (or bulging) testes. F1s of Oregon-R flies crossed to w*; Actin5C-GAL4/CyO were used as control. “*”*p* < 0.05, ‘**’*p* < 0.01.

**Figure 2:**
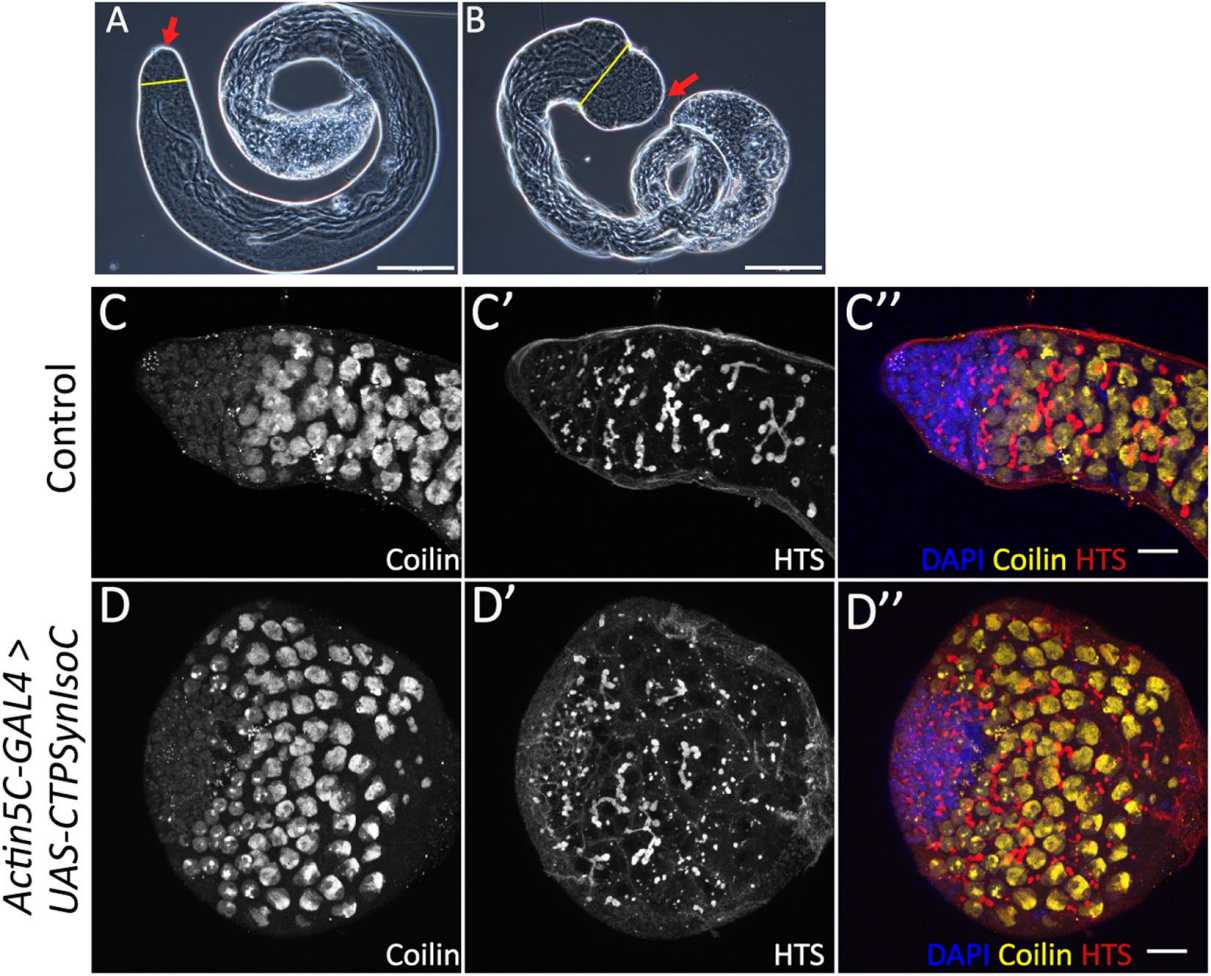
Diagrams showing control testis and transgenic testis. (**A**) Phase contrast image of a control testis. (**B**) Phase contrast image of *Actin5C-GAL4* > *UAS-CTPSynIsoC* testis showing the bulging phenotype. Red arrow indicates apical tips of the testes. Yellow line shows the relative diameter of the testis body. Scale bar: 200 µm. (**C**–**C”**, **D**–**D”**) Confocal images showing the control testis and bulged testis stained with anti-Coilin to label secondary spermatocytes. Scale bar: 20 µm. Side panels show genotype.

We first identified the variations in phenotypes that *CTPSynIsoC* would cause in testicular tissue when it is expressed under the influence of a strong, ubiquitous *Actin5C* driver. All males of selective genotype were dissected. Testes were observed under the microscope. ‘Bulging’ was defined as a visibly significant increase in circumference across the apical tip when compared to the control genotype (*Actin5C-GAL4* > *Oregon-R*). A schematic representation of a normal testis and early spermatogenesis is represented in Fig. 1A. About 34% (n = 525, Fig. 1B) of *Act5C-GAL4* > *UAS-CTPSynIsoC* testes were overgrown, as shown in Fig. 2B. Preliminary staining study of testes using anti-Coilin to label secondary spermatocytes showed a higher number of Coilin-positive cells in the transgenic, bulging testis (Fig. 2C–D”).

**Figure 3:**
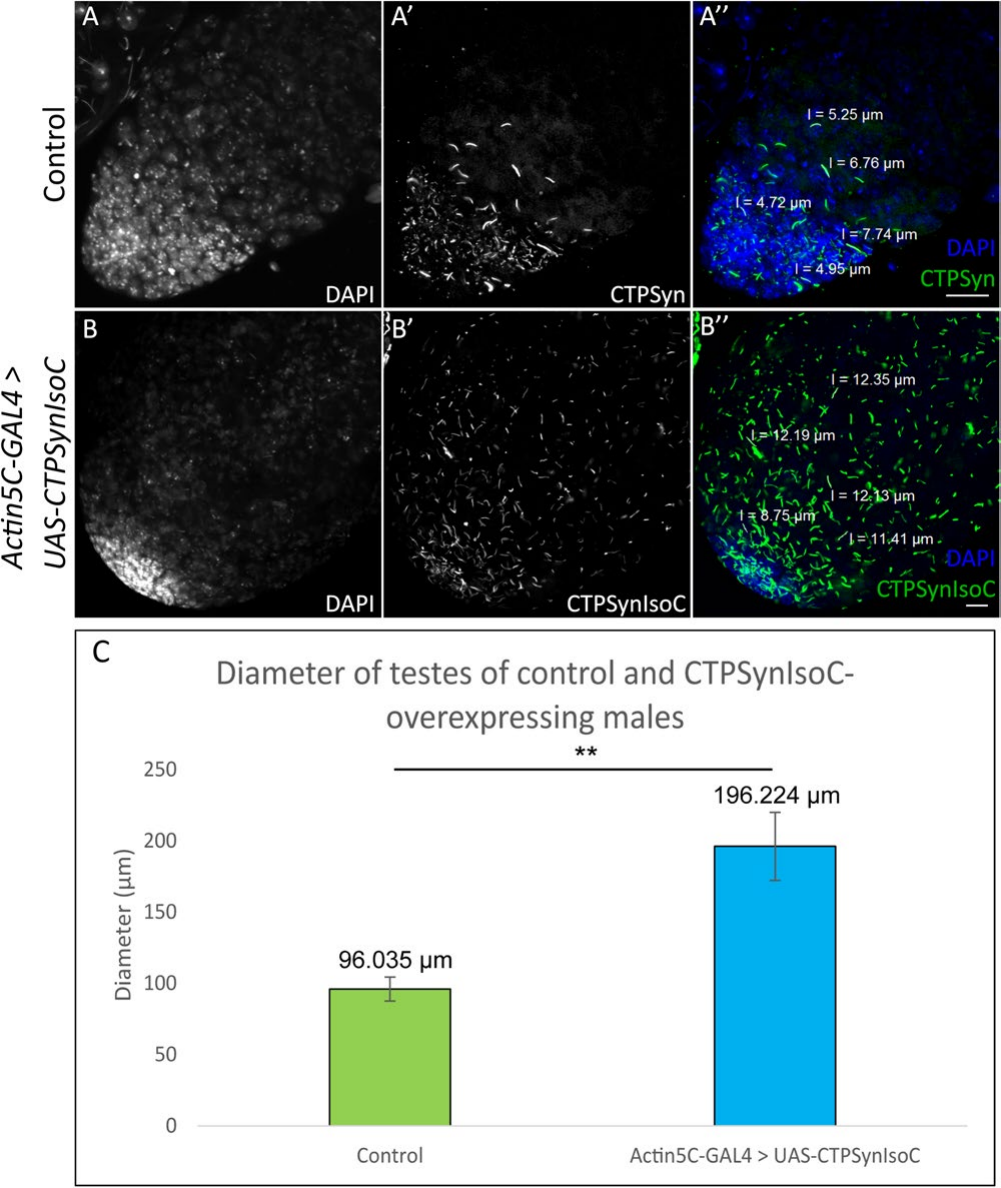
*CTPSynIsoC* overexpression induced longer cytoophidia formation. (**A**–**A”**) Confocal images showing control testis stained with anti-CTPSyn. (**B**–**B”**) *CTPSynIsoC*-*Venus* tagged overexpression testis causing the bulging phenotype. Cytoophidia length in control testis were shorter compared to *CTPSynIsoC* overexpression testis. Scale bar: 20 µm. Side panels show genotype. (**C**) Bar chart showing average diameter (µm) of the testicular width from control and transgenic testes. “**”*p* < 0.01. (See Supplementary Fig. 2 for confocal images).

To show that this bulging phenotype could indeed be related to the overexpression of *CTPSynIsoC*, fixed testes were stained against CTPSyn (Fig. 3A–B”). Cytoophidia morphology and localization patterns seen in controls (*Actin5C-GAL4* > *Oregon-R*) were consistent to descriptions in a previous report^10^. Both micro and macro-cytoophidia were present in these tissues. Whereas greater concentrations of micro-cytoophidia were observed towards the end of the transit amplification (TA) region, macro-cytoophidia are often found in a more random, dispersed pattern across the same foci. No cytoophidia were detected beyond this region. Conversely, not only do bulged testes from the transgenic males (*Actin5C-GAL4* > *UAS-CTPSynIsoC*) show lengthened cytoophidia across the apical tips, the structure is preserved in cells of later stages of spermatogenesis as well. The correlative incidence percentage between testicular bulging and cytoophidia elongation in these cases was 100%. Bulged testes and control testes were measured to demonstrate the differences in diameter as shown in Fig. 3C. The diameter of apical tips of *CTPSynIsoC*-overexpressing testes (196.22 µm) increased two-fold compared to control testis (96.04 µm) (confocal images are shown in Supplementary Fig. 2).

### Apical testes bulging arose from increased germline/spermatocyte numbers

**Figure 4:**
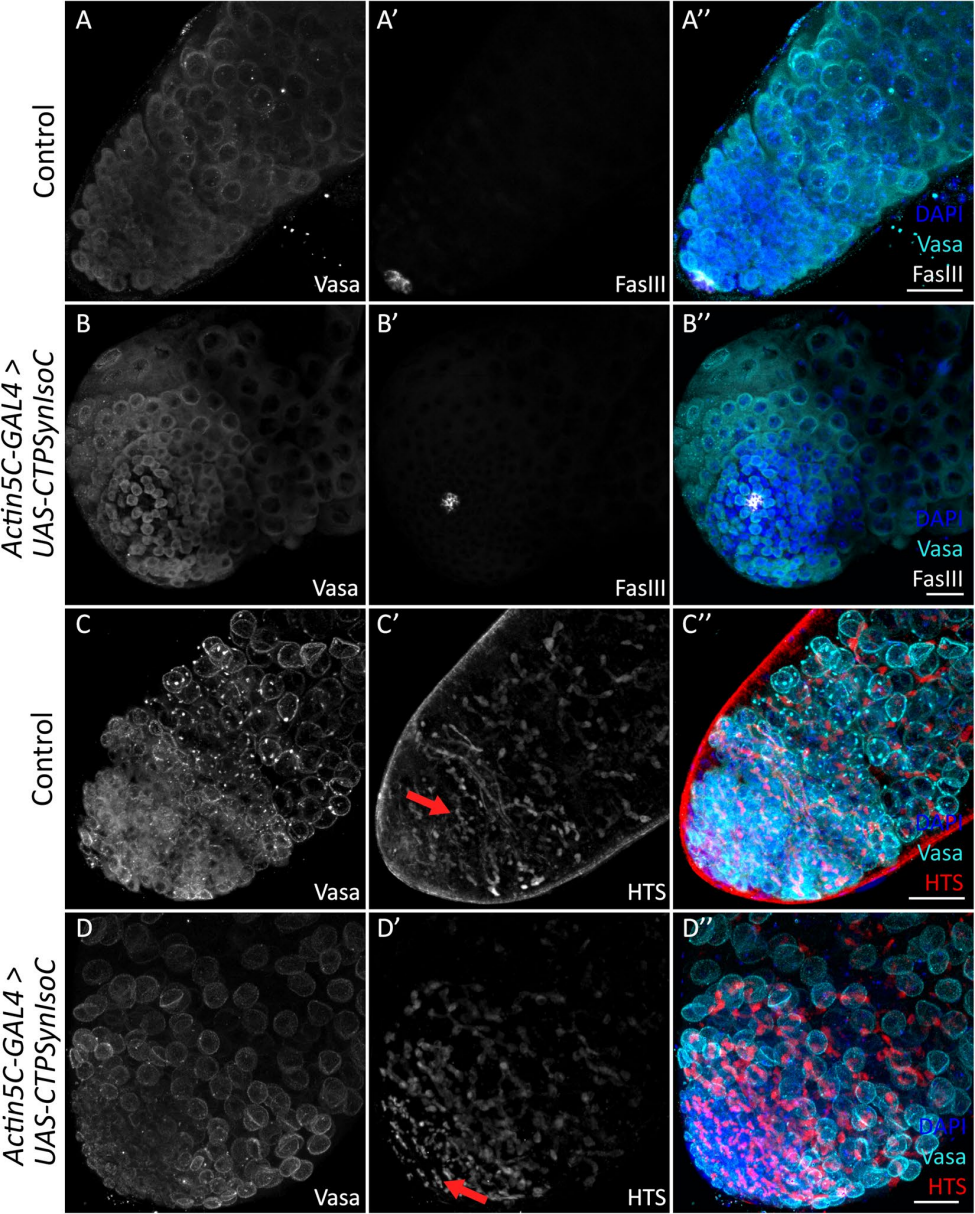
Testes stained with Anti-Vasa, Anti-FascIII, and Anti-HTS. (**A**–**A”**) Control testis and (**B**–**B”**) *Actin5C-GAL4* > *UAS-CTPSynIsoC* testis stained with anti-Vasa and anti-FasIII. Note that the numbers of GSC adjacent to the hub cells are phenotypically normal in *CTPSynIsoC*-overexpressing testis (**B’** and **B”**). (**C**–**C”**) Control testis and (**D**–**D”**) *Actin5C-GAL4* > *UAS-CTPSynIsoC* testis stained with anti-Vasa and anti-HTS. The branching of fusome occurs relatively early in *CTPSynIsoC*-overexpressing males. Scale bar: 20 µm.

We next attempted to identify the cause of testicular bulging through immunohistochemistry methods, refined to stain against certain markers of *Drosophila* germline stem cell (GSC) and cyst stem cell (CySC) progression. These two cell-types are bound in to the somatic hub cells (stained by Anti-FascIII) in a rosette-formation. Together, this point in the apical region of the testis is known as the stem cell niche. GSCs and CySCs divide to give rise to spermatogonia and spermatogonium-enveloping cyst cells, respectively (Fig. 1A). Anti-Vasa highlights GSCs as well as germline cells, whereas Anti-Tj (Traffic-Jam) stains both CySCs and their immediate descendants. A larger population of Vasa-positive cells was observed in *Act5C-GAL4* > *UAS-CTPSynIsoC* testes compared to controls, suggesting CTPSyn-influenced growth in germline cell population (Fig. 4A,C vs 4B,D). GSCs themselves were however not affected by the overexpressed protein and remained as an eight-cell rosette (Fig. 4B,B’). We subsequently stained fusome with Anti-HTS (hu-li tai shao)^25^. Fusome is a germline specific organelle. As germline cells develop, the morphology of fusome changes from spherical in GSC to interconnected branches in spermatogonium and its descendants due to incomplete cytokinesis^26^. In control testes, the branching of fusome is observed at a certain distance away from the apical tip. In the *Actin5C-GAL4* > *UAS-CTPSynIsoC* flies, not only does branching become apparent earlier i.e. in cells closer to the stem cell niche, these structures are found in greater numbers than in controls (Fig. 4C’,D’, red arrow). This shows that germline cells in *CTPSynIsoC-*overexpressing testes not only divide faster, but also divide more, producing larger populations of growing cells.

**Figure 5:**
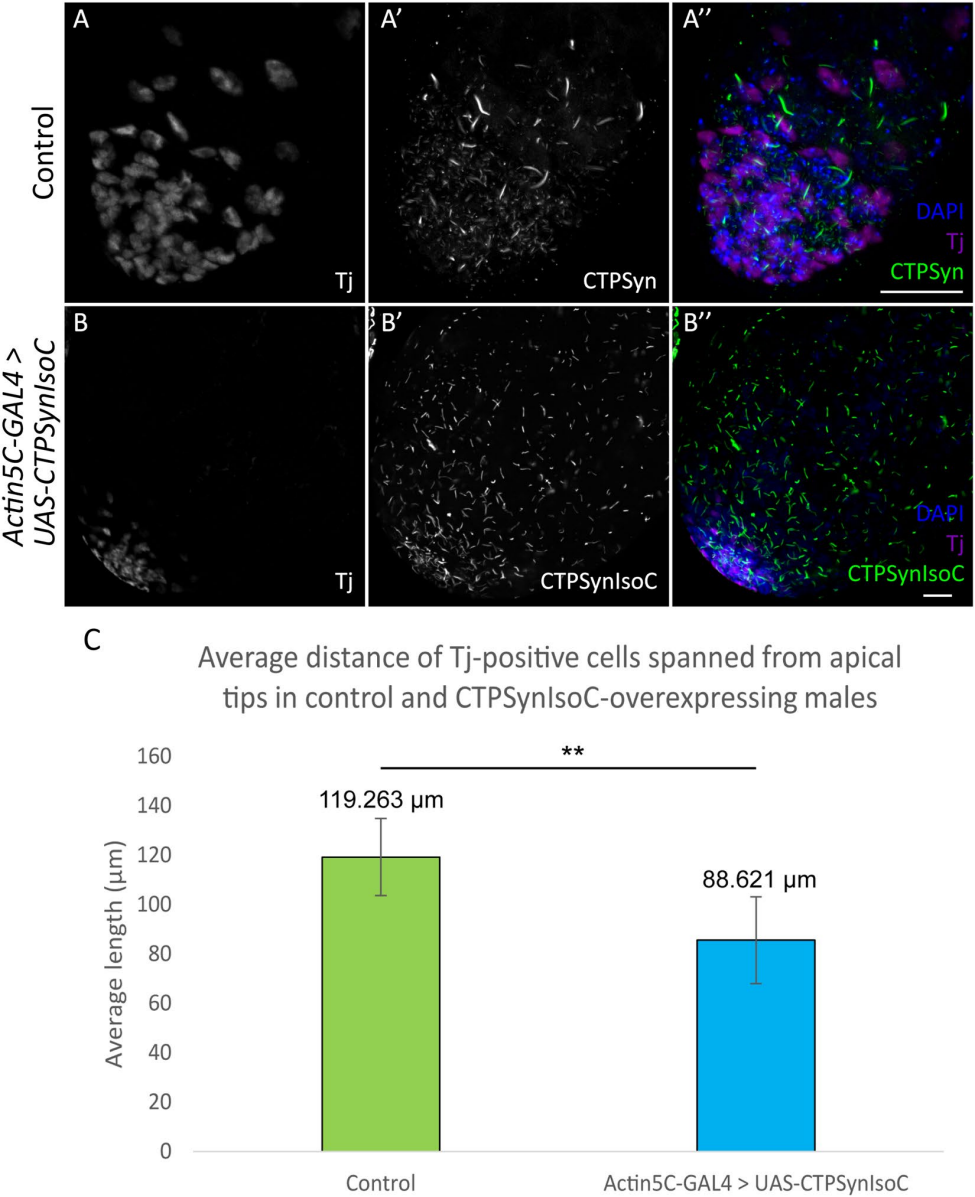
Testes stained with anti-Traffic jam (Tj) and anti-CTPSyn. (**A**–**A”**) Control testis and (**B**–**B”**) *Actin5C-GAL4* > *UAS-CTPSynIsoC* testis stained with anti-Tj and anti-CTPSyn. The CySC and early cyst cells (Traffic jam) were found to be loosely spaced in control testis, while the Tj-positive cells were more clustered and closely packed in transgenic testis. Scale bar: 20 µm. (**C**) Bar chart showing average length of Tj-positive cells spanned from apical tip of testis in both control and transgenic group. **p < 0.01. (See Supplementary Fig. 3 for confocal images).

**Figure 6:**
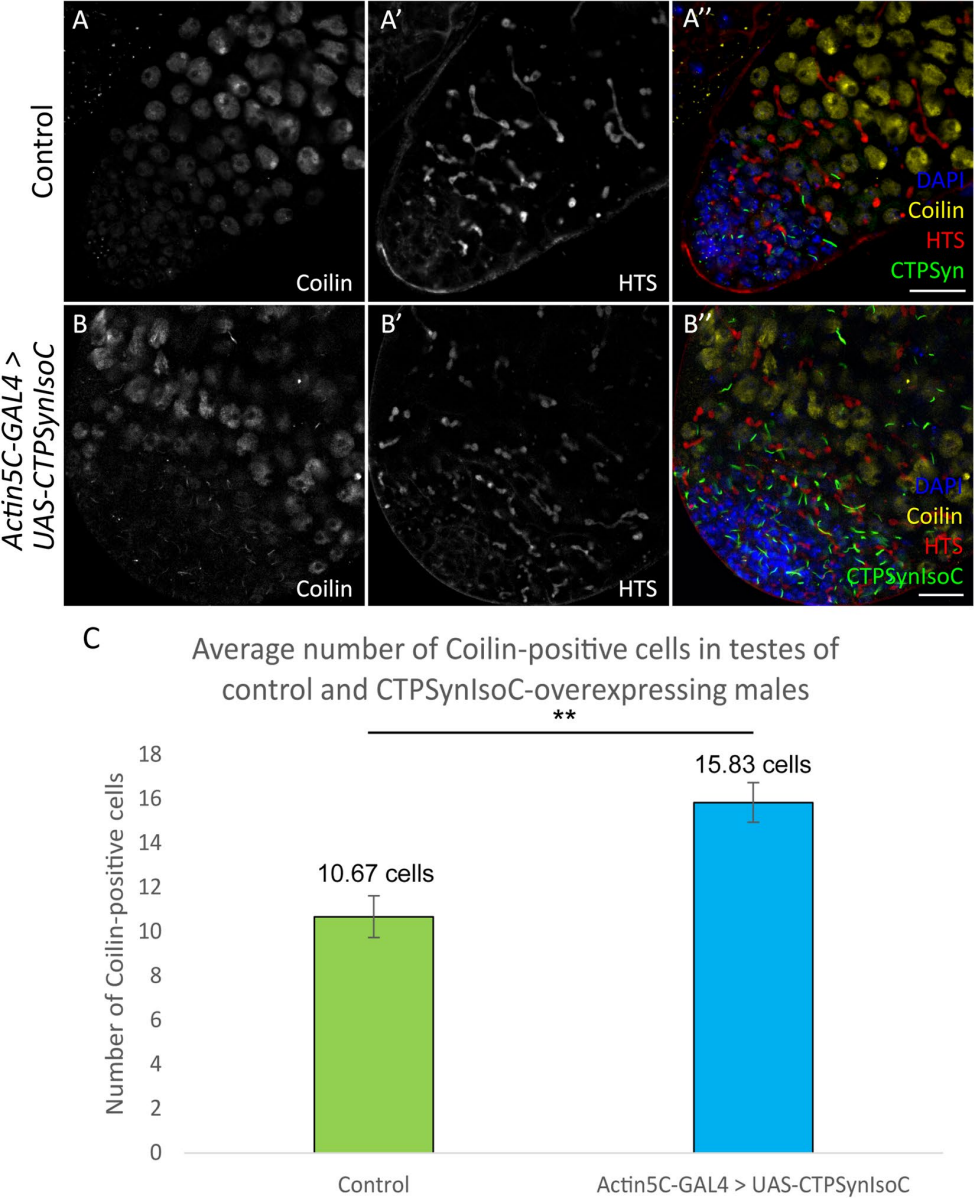
Testes stained with anti-Coilin and anti-HTS. (**A**–**A”**) Control testis and (**B**–**B”**) *Actin5C-GAL4* > *UAS-CTPSynIsoC* testis stained with anti-Coilin and anti-HTS. Number of Coilin-positive cells increased across the width of the transgenic testis compared to control testis. Note that single plane from z-stack images were used instead of maximum projection. Scale bar: 20 µm. (**C**) Bar chart showing the average number of Coilin-positive cells across the width of testes. **p < 0.01.

**Figure 7:**
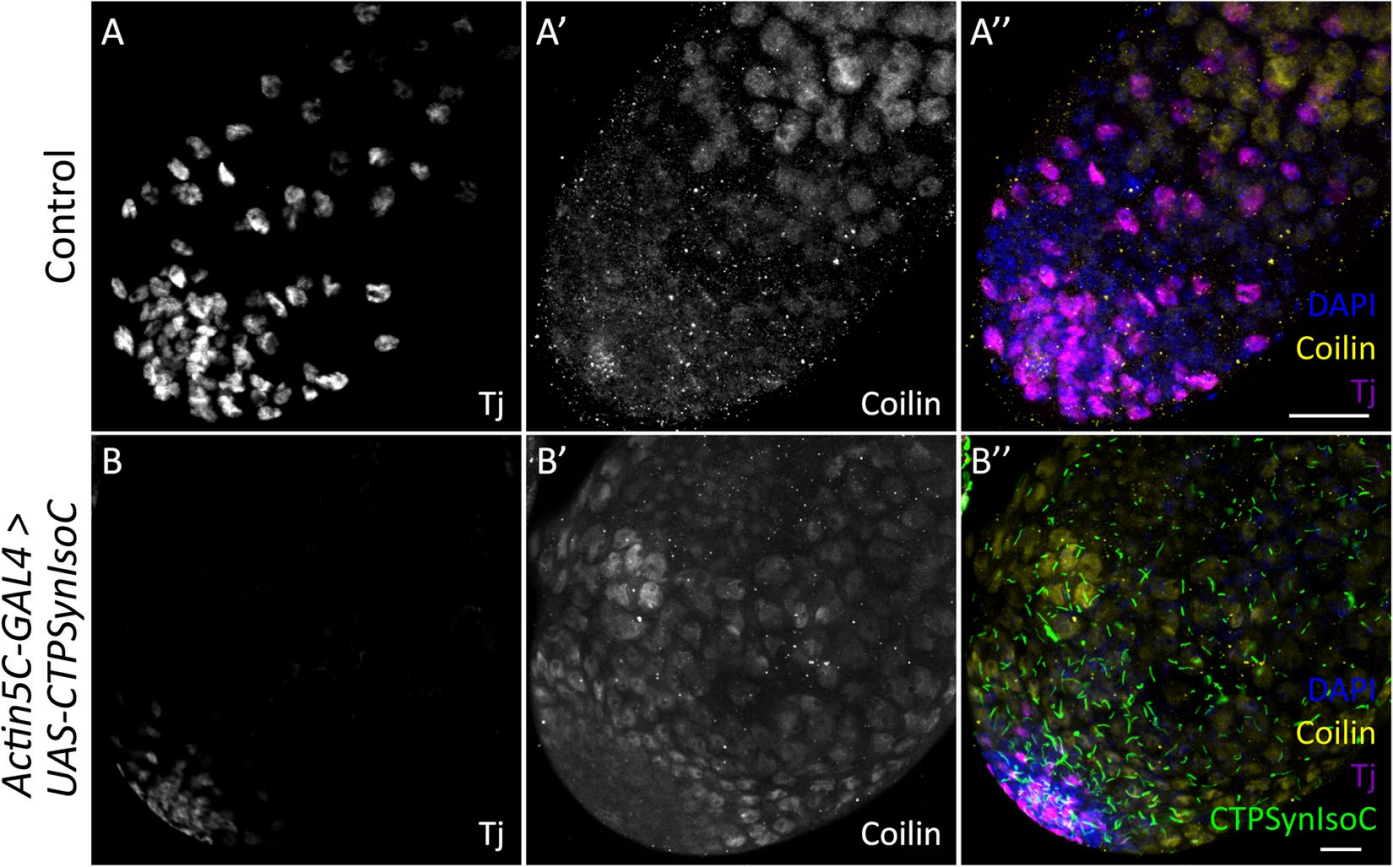
Staining showing testes with anti-Traffic jam and anti-Coilin. In control testis (**A**–**A”**), Tj-postive cells were found to be loosely spaced, followed by upregulation of Coilin. In transgenic testis (**B**–**B”**), Tj-positive cells were clustered and closely packed. Note that (**B**–**B”**) is a single plane image instead of maximum projection. Scale bar: 20 µm.

Given the relationship of germline cells to somatic cyst cells, an increase in Vasa-positive cell numbers should be accompanied by increased cyst cells, which are labelled by anti-Tj. However, the opposite was instead shown to be true: In control testes, Tj-positive cells were loosely spaced and found well into the testicular trunk. In *Actin5C-GAL4* > *UAS-CTPSynIsoC* flies, however, such cells are found only in regions close to the apical tip, clustered around GSCs (Fig. 5A,B). We found that the distance of Tj-positive cells spanned from the hub cells varied in both control and *CTPSynIsoC*-overexpressing testes. In control testes, the Tj-positive cells spanned across an average distance of 192.26 µm compared to 88.62 µm in the bulged testes (Fig. 5C, see Supplementary Fig. 3 for confocal images). To further document the properties of germline descendants, we stained for Coilin. A GSC divides asymmetrically to give rise to a daughter gonialblast (GB). After four rounds of transit amplification divisions, a spermatogonial cyst comprising of 16 cells is produced. These will later mature into spermatocytes. As Coilin is up-regulated in spermatocytes^27^, an increase in Vasa-positive cell numbers should be accompanied with increased numbers of Coilin-positive cells. We observed higher numbers of such cells across the width of *Actin5C-GAL4* > *UAS-CTPSynIsoC* testes, compared to the control testes (Fig. 6A–6B”). By counting the Coilin-positive cells across the apical tips of both transgenic and control testes, we found that the average number of Coilin-positive cells were 16 and 11 cells, respectively (Fig. 6C, see Supplementary Fig. 4 for confocal images). These findings together show that the ubiquitous overexpression of *CTPSynIsoC* within testicular tissue does indeed induce germline cell proliferation and differentiation, leading to higher cell counts and its bulging phenotype. It must be noted that though Coilin-staining remained strong in the testicular trunk of *CTPSynIsoC-*overexpressing testes, Tj-positive cells were constrained to the apical tip (Fig. 7A–B”). When taken together with Vasa-staining patterns, these observations affirm further that rather than spermatocytes, CySCs and its descendants develop alongside early germline cells instead.

### MiR-975 causes similar levels of apical testis bulging and increase of spermatocyte cells in males overexpressing the microRNA

**Figure 8:**
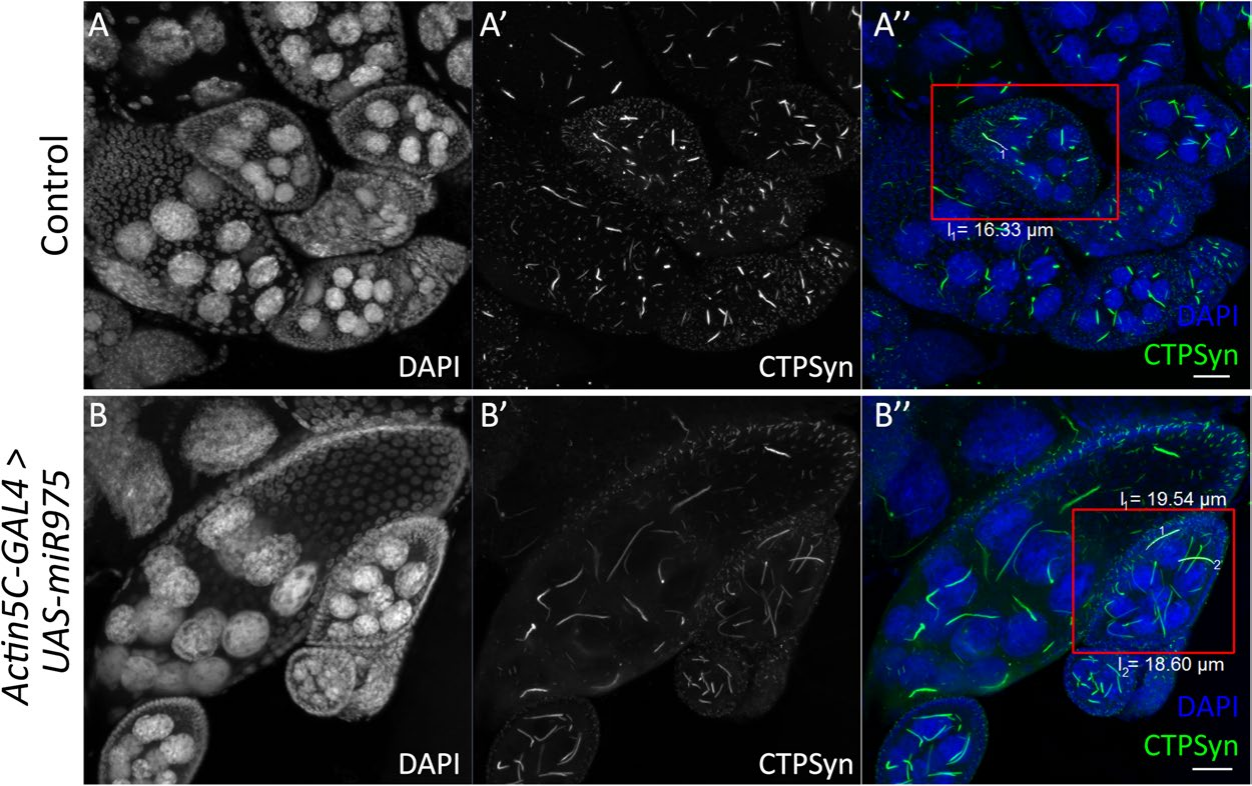
Overexpression of miR-975 induces cytoophidia elongation in egg chambers. (**A**–**A”**) Cytoophidia in control ovaries and (**B**–**B”**) *Actin5C-GAL4* > *UAS-miR-975* ovaries. Stage 8 egg chambers are highlighted in red boxes. Note the elongated cytoophidia in **B’** and **B”**, which were longer than the long cytoophidia found in **A’** and **A”**. Scale bar: 20 µm.

A parallel screening for alterations in cytoophidia phenotypes caused by the overexpression of singular microRNAs (miRNA) was conducted along with our investigation into *CTPSynIsoC* overexpression^28^. Lines carrying miRNA genes were crossed to various drivers. The morphology of cytoophidia in the ovaries of emergent F1 females was then observed. We saw that flies overexpressing miR-975 displayed significantly lengthier cytoophidia in the nurse cells of their egg chambers, regardless of driver-construct (Fig. 8B, vs 8A i.e. control). Cytoophidia within the follicle cells of *T155-GAL4* > *UAS-miR-975* individuals, however, were not affected (data not shown). Upregulation of *CTPSynIsoC* in ovarian RNA extracts were confirmed via qPCR.

**Figure 9:**
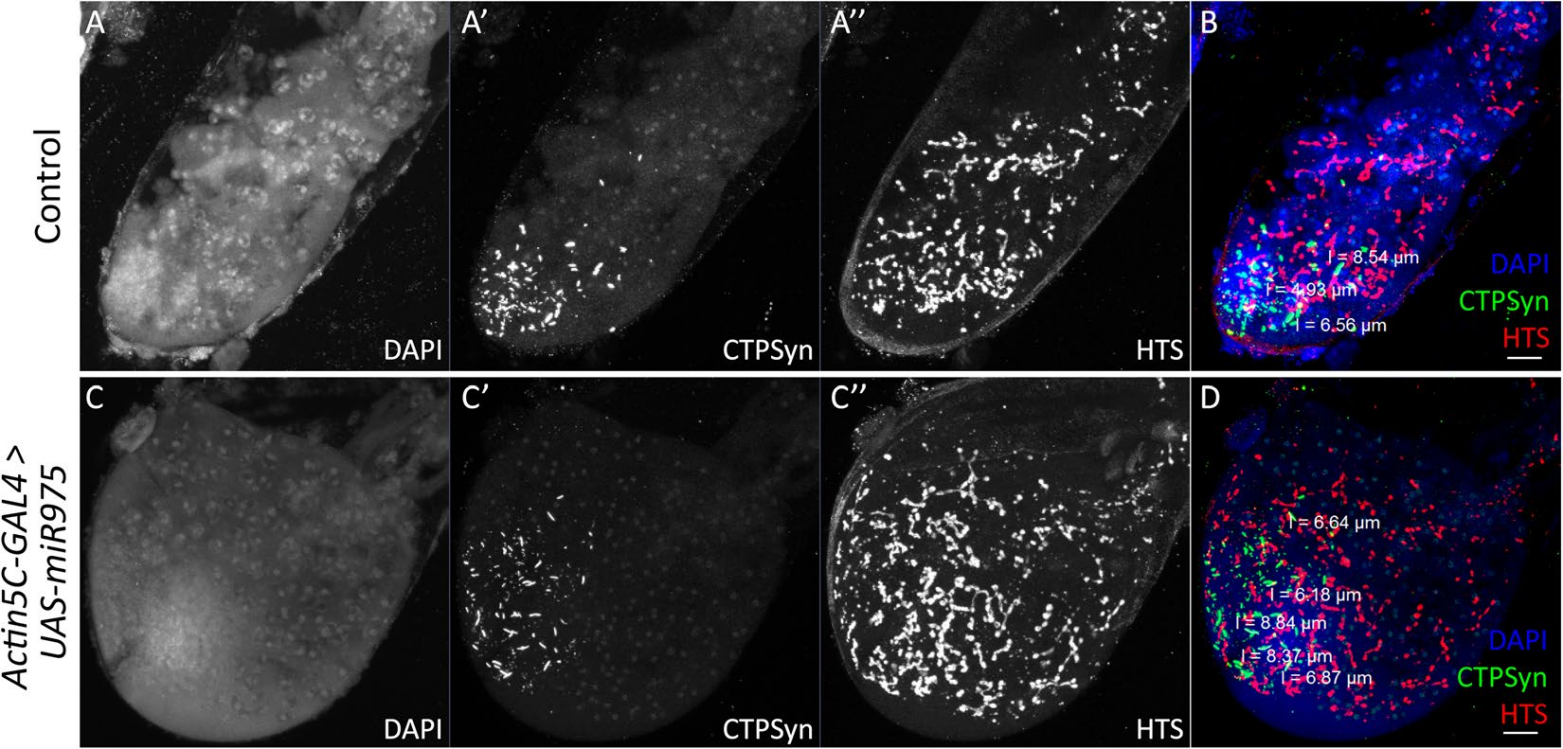
Increased numbers of bulged testes are recovered in males overexpressing miR-975. (**A**–**A”**) Control testis, merged shown as (**B**). (**C**–**C”**) *Actin5C-GAL4* > *UAS-miR-975* testis, merged shown as (**D**). Cytoophidia length in miR-975-overexpressing testis did not elongate significantly as compared to control testis. Scale bar: 20 µm.

We hypothesized from this information that as *CTPSynIsoC* levels are positively affected by the overexpression of miR-975, like-genotyped males will likely display the same apical bulging phenotype as observed in those overexpressing *CTPSynIsoC*. This was found to be true. Figure 9 clearly shows a circumferential difference between *Actin5C-GAL4* > *Oregon-R* and *Actin5C-GAL4* > *UAS-miR-975* males. These images further demonstrate a marked increase in fusomic-branching incidences in flies of the latter genotype. Unlike *Actin5C-GAL4* > *UAS-miR-975* females, however, neither cytoophidia length nor numbers appeared affected (Fig. 9B–D). Instead, a noticeably greater presence of nuclear CTPSyn – rather than the protein in its tethered form as cytoophidia – was seen beyond the stem cell-niche region in testes tissue overexpressing miR-975. Phenotyping additionally show that these flies would produce similar penetrance levels of bulged testes to that of *Actin5C-GAL4* > *UAS-CTPSynIsoC* males (Fig. 1B).

### Overexpression of either *CTPSynIsoC* and miR-975 lead to upregulated levels of isoforms of *CTPSyn*, but show differential association with commonly-linked genes

**Figure 10:**
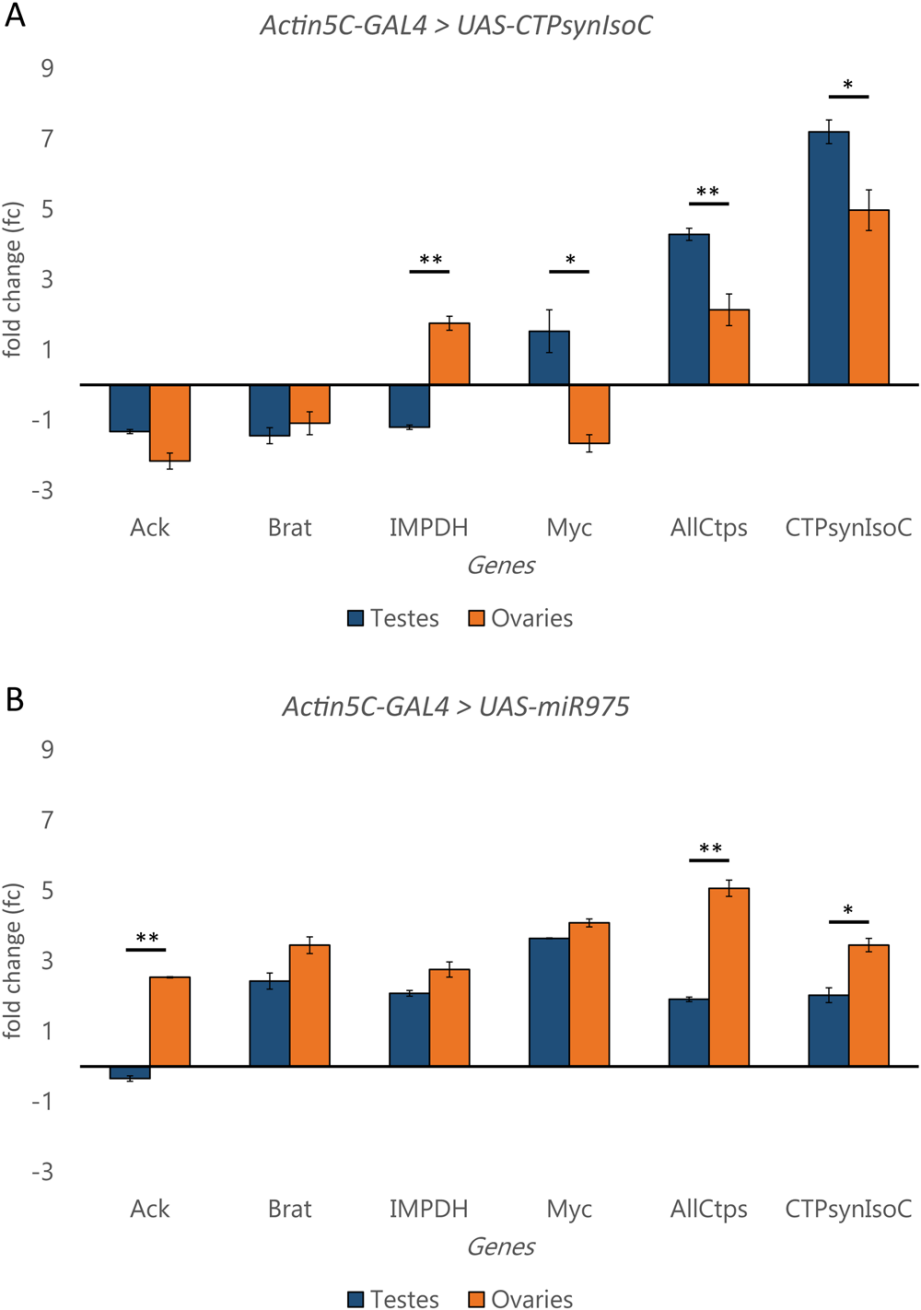
Overexpression of *CTPSynIsoC* and miR-975 each affects expression levels of *CTPSyn*-related oncogenes differently. (**A**) Overexpression of *CTPSynIsoC* affects *IMPDH* and *Myc* differently in reproductive tissues of different sexes. In (**B**), miR-975 overexpression only induced such opposite response of the *Ack1* gene. A statistically significant change is marked with ‘**’, where *p* < 0.01.

Tissue overgrowth is a characteristic hallmark of tumours. It is thus natural to speculate that the bulging testes phenotype observed with either *CTPSynIsoC* or miR-975 overexpression is attributable to known oncogenic factors. We narrowed a candidate pool down to four genes, all of which have either shown a connection to testicular or prostate cancer in past literature, and/or a demonstrated relationship to *CTPsyn*^18,19,21^. The expression levels of these genes in testes tissue from *Act5C-GAL4* > *UAS-CTPSynIsoC* were compared to their expression levels in *Actin5C-GAL4* > *UAS-miR-975*. *Rp49* and *GAPDH* served as references for normalization purposes. Fold-change (fc) values were derived after discounting expressional fluctuations observed in the *Actin5C-GAL4* > *Oregon-R* controls, where fc is assumed to be zero for each gene of interest (Fig. 10).

As expected, *Actin5C-GAL4* > *UAS-CTPSynIsoC* showed a drastic increase in *CTPSynIsoC* levels (fc = 7.20). The primer pair for *AllCtps* was designed to simultaneously capture Isoforms A, B and C of *D*. *melanogaster* *CTPSyn* (*dmeCTPSyn*). A 4.28-fold upregulation of these isoforms was recorded in *Actin5C-GAL4* > *UAS-CTPSynIsoC* flies. Synergy amongst isoforms is well-documented, and thus this observation was anticipated. However, as we are unable to distinguish isoforms from one another here, the ratios in which each isoform would have contributed to the fold-change would also remain unclear. Nonetheless, as the *Actin5C-GAL4* > *UAS-CTPSynIsoC* crosses are expected to drive the expression of *CTPSynIsoC* alone, it is likely that the upregulation is attributable to the much higher numbers of this isoform, rather than a positive change in any of the others.

We had expected an upregulation of *CTPSynIsoC* in *Actin5C-GAL4* > *UAS-miR-975* males as well. This was a hypothesis inferred from the cytoophidia-elongating effect that the overexpression of this particular miRNA had on these filamentous structures when they were observed in female egg chambers, and the supporting confirmation that *CTPSynIsoC* was indeed upregulated in ovarian extracts of these flies. We observed moderate upregulations in both *CTPSynIsoC* (fc = 2.02) and *AllCtps* (fc = 1.91). Compared to *Actin5C-GAL4* > *UAS-CTPSynIsoC* testes – where the fc disparity between these two were very much obvious, while their differences in *Actin5C-GAL4* > *UAS-miR-975* testes were minute (*p* > *0*.*05*). This allows us to deduce that rather than *CTPSynIsoC*, the upregulation of CTPSyn seen in *Actin5C-GAL4* > *UAS-miR-975* testes is attributable in a much more equally-distributed manner amongst the three isoforms.

*Actin5C-GAL4* > *UAS-CTPSynIsoC* testes demonstrated downregulations in *Ack*, *Brat* and *IMPDH*, whilst showing an upregulation in *Myc*. However, fc values were modest across all four genes; none exceeded an fc of 1.6 (fcs = −1.32, −1.44, −1.20, and 1.53, respectively). Fluctuations seen in *Myc* readings between bio-replicates discounts the significance of its upregulation as a causal factor for testes bulging in *Actin5C-GAL4* > *UAS-CTPSynIsoC* even more. By contrast, *Actin5C-GAL4* > *UAS-miR-975* testes displayed positive correlations to *Brat*, *IMPDH* and *Myc* (fcs = 2.42, 2.08 and 3.64, respectively). This indicates that at the very least, excessive levels of the miRNA may be related to the activation of several cancer-related factors. Although upregulations of these cancer-related factors were observed, the underlying mechanisms of these genes, both directly and indirectly, which lead to germline cells expansion thus the bulging phenotype when *miR-975* was overexpressed still remain exclusive.

## Discussion

The *Drosophila* testis is a close-ended muscular tube sheathed by pigment cells and acto-myosin of genital disc origin^29^. Here, paralleled division between GSCs and CySCs gives rise to units of gonialblasts (GBs) surrounded by daughter cyst cells^30^. Due to the highly-coordinated manner in which this event occurs, cell-to-cell signalling across the niche microenvironment is vital. We have demonstrated how males overexpressing *CTPSynIsoC* have a much greater tendency to produce bulged testes. Tissue overgrowth is always exclusive to the apical tip, where the stem cell niche resides and associated intercellular communication events transpire. In such situations where it is found in excess, is *CTPSyn* then capable of disturbing the delicate balance observed between these signalling molecules, leading to uncontrolled cell proliferation? Several cancer cell lines of hepatoma^16^, leukaemia^17,31–33^ and colon^34^ origin are known to intrinsically express higher levels of CTPSyn. Conversely, a more recent study has demonstrated how RNAi-mediated knockdown of the gene could reduce tumour overgrowth significantly in *Drosophila* larvae^35^. With this study, we ask whether the overexpression of *CTPSyn* alone could generate tissues or organs with cancerous properties.

In the *Drosophila* testis, each GB undergoes four rounds of transit amplification (TA) divisions. Rapid production of nascent nuclei within such a limited space means that this region outwardly appears dense and tightly-packed under DNA-counterstaining by DAPI. In *Actin5C-GAL4* > *Oregon-R* controls, greater incidences of micro-cytoophidia is recorded here as well. Beyond the TA region, macro-cytoophidia are typically seen instead. These observations suggest a flexibility in cytoophidia formation, even across relatively small distances. We hypothesize that this interchangeable nature of CTPSyn filamentation might be both a means of regulating the protein’s activity, and a mechanism to modulate response to cellular needs for its CTP product. As aforementioned, the TA region is where active mitotic division occurs continuously. This creates an environment of high-nucleotide demand. The formation of cytoophidium has been shown to signify the inactivity of *dme*CTPSyn^36^. High CTP demand calls for greater enzymatic activity; more and more CTPSyn proteins thusly dissociate from its filament form, gradually causing a substantial shortening of cytoophidia. CTPSyn is also involved in the synthesis of phospholipids^37^, another component required during the cytokinesis process following nuclear separation in mitosis. Vasa and Coilin staining together further show that in comparison to controls, *Actin5C-GAL4* > *UAS-CTPSynIsoC* testes do indeed bear higher numbers of dividing GSCs close to the stem cell niche, accelerating downstream spermatocyte population growth. With its functions so closely tied to mitotic cellular division, it is no wonder then that uncontrolled levels of CTPSyn proteins appear to single-handedly orchestrate over-proliferation events within the TA region, leading to apical tip bulging.

Similar to *CTPSynIsoC*-overexpression, initial screening phases into identifying cytoophidia-altering miRNA overexpression events saw us exclusively scrutinizing ovarian cells as well. Our choice to do so was also in part due to limitations imposed by expressional characteristics of employed GAL4-drivers. For most miRNAs, we were doubly limited by the purported inadequacy of the UASt construct within germline cells^38^. Regardless, concurrent utilization of both follicle-cell and nurse-cell capable drivers allowed us to effectively isolate a small group of candidate miRNAs by their effects on cytoophidia morphology^39^.

Our miRNA-overexpression screen revealed that when expressed in excess, miR-975 asserts an elongating effect upon ovarian cytoophidia. We now know that observed elongation in females could translate into testicular apical tip enlargement in approximately 20% of emergent males. Though less extreme than the *Act-GAL4* > *UAS-CTPSynIsoC* phenotype, the lengthening of cytoophidia was still prominent enough in *Actin5C-GAL4* > *UAS-miR-975* females for us to be optimistic about obtaining bulged testes in like-genotyped males. RT-qPCR further confirmed a modest upregulation of *CTPsynIsoC* in testes overexpressing miR-975.

Subsequent phenotyping revealed that not only are *Actin5C-GAL4* > *UAS-miR-975* males producing bulged testes, but a significantly high proportion of them do (20.53%; n = 219). Of course, the relationship between microRNAs and cancer is well studied^40^. The critical roles they play in monitoring gene expression often mean that with their dysregulation, the disruption to cellular ‘normal’ which typically follow may eventually lead to cancerous phenotypes. Many miRNAs mimic tumour suppressors in functionality^41^. An inverse relationship with carcinogenesis is thusly expected in most cases. Several miRNAs are nonetheless known to encourage the progression of certain cancer types such as lung and hepatocellular carcinoma, rather than repressing them^42,43^. The trio-clustered *hsa*miRNAs 371, 372, and 373 have proven themselves to be potent oncogenes. Their part in driving testicular germ cell tumours in particular is widely acknowledged, so much so that measuring sera hsa-miR371 levels is now applied as a diagnostic biomarker tool^44^.

In this study, the overexpression of miR-975 alone appears to have sufficiently induced visible tissue overgrowth in *Drosophila* testes. As with *CTPSynIsoC*-overexpressing flies, we endeavoured to understand how this phenotype could have been caused by staining bulged testes with anti-HTS. The primary antibody stains for Adducin-like proteins, found within fusomes. These proteins are divided between two nascent fusome organelles during cell-division. However, due to the incomplete nature of cytokinesis amongst dividing spermatogonial cells, fusomic-division is by consequence also incomplete; Adducin-like proteins are thus highlighted as thin structures inter-bridging neighbouring spermatogonia within developing cysts. These are referred to as ‘fusomic-branches’^45^. Figure 9 demonstrates that in comparison to control, miR975*-*overexpressing testes show higher incidences of fusomic-branching. This overlaps patterns seen with anti-HTS staining in *CTPSynIsoC-*overexpressing males. As fusomes are temporally seen as early as within gonialblasts (GB), we can conclude that akin to *Actin5C-GAL4* > *UAS-CTPSynIsoC*, testes bulging in *Actin5C-GAL4* > *UAS-miR-975* too is attributable to excessively robust GB production around the stem cell niche. Girth-expansion may therefore be a result of the tissue accommodating above-normal spermatocyte population sizes. In our opinion, the highly positive association miR-975 overexpression has with an upregulation of cancer-driving genes such as *IMPDH* and *Myc* only strengthens its case as a potential onco-miR. However, there are no studies describe the direct effects of the miR-975 on tumorigensis; while in this study, miR-975 was shown to be contributing to germline cells expansion instead.

Nonetheless, *Brat* expression levels was also positively affected by its overexpression where we had conversely expected its downregulation, due to its role as a tumour suppressor. Functionally, *Brat* is powerful enough to markedly decrease *dme*Myc protein levels in various *Drosophila* tissues^46^. We can only speculate that the compounding effects of *Myc* and *IMPDH* upregulation, as positive drivers of cell growth, trumped any down-regulatory effects *Brat* may have as a tumour suppressor. We have also reiterated the importance of considering the possibility of sex-disparate phenotypes in GSCs time and time again within this study: whereas the relationship between Brat and *dme*Myc has been demonstrated in females^47^, it has yet to be described for males. Finally, due to the fact that *miR-975* overexpression increase the levels of CTPSyn, and overexpression of *CTPSynIsoC* causes the bulging phenotype, it is possible that CTPSyn plays a significant role in cell proliferation regulations, which by itself or with the combination of Myc, causes the phenotype. Further investigations involving detailed transcriptome data comparison between female and male GSCs should provide a more in-depth understanding.

## Conclusions

In this study, we demonstrate that *CTPSynIsoC* overexpression causes a lengthening of cytoophidia in testes tissue. For a third of males, this culminates in the form of apical tip bulging. Higher numbers of germline cells and spermatocytes is shown to be main cause of testicular overgrowth, as these are stained by Anti-Vasa and Anti-Coilin, respectively. A miRNA i.e. miR-975 is also found to induce the bulged-testes phenotype in ~20% of males overexpressing it, although cytoophidia length, size, and compaction is not visibly altered. RT-qPCR nonetheless reveals that a significant increment in *CTPSynIsoC* levels does accompany miR-975 overexpression. This is not a two-way relationship, *CTPSynIsoC* overexpression does not cause miR-975 upregulation. Quantification of several cancer-related genes further show how differentially either overexpression events affect genetic expression, suggesting that *CTPSyn* might be closely related to these genes, if not at least by parts. They both also affect *Myc* expression positively. As a potent oncogene, it is possible that the defining induction event in testes overexpressing either *CTPSynIsoC* or miR-975 is Myc-protein production, the combination effects of which would have eventually led to excessive proliferation, and therefore tissue-overgrowth. Though the findings presented here are insufficient to solidly link *CTPsyn* via miR-975 to tumorigenesis, this study has demonstrated that the overexpression of either gene could affect tissue growth in such a way that it culminates a germline cells hyper-growth. For this reason, we also propose greater utilization of testes as a source of germline cells and its stem cells niche studies. In the future, simultaneous overexpression of *CTPSynIsoC* and miR-975 could be employed to further investigate if there is a connection between the enzyme and the miRNA. Knock-down experiments using RNAi and transcriptomics as well as RNA sequencing will also be implemented to identify the causes of the bulging phenotype, and to clarify once and for all whether these underlying mechanisms are indeed cancerous by nature.

## Materials and Methods

### Fly stocks

Stocks were raised at 25 °C on cornmeal; the recipe has been slightly modified to suit the local climate and availability of ingredients. The sole driver-GAL4 line involved in this study is *Actin5C-GAL4/CyO* (Kyoto *Drosophila* Genome and Genetic Resources (KGGR) #107727). Progenies of *Actin5C-GAL4/CyO* crossed to Oregon-R flies (we refer this line as *Actin5C-GAL4* > *Oregon-R* in this article) were controls for all experiments unless stated otherwise^48,49^. *UAS-CTPSynIsoC* was a gift from the Liu lab in DPAG, University of Oxford^12^. The expression of this construct produces Venus-tagged CTPSynIsoC proteins. *UAS-miR-975* lines were obtained from Bloomington Stock Center (Bloomington #60663) and Institute of Molecular and Cell Biology, Singapore (genotype *miR-975@VK37/CyO* and *miR-975@86Fb*), a gift from Stephen Cohen. Crosses made were grown at 28 °C in a 12 h/12 h light to dark cycle.

### Immunohistochemistry

F1 flies were flipped onto wet yeast. Males aged 5 to 6 DAE were dissected in ice-cold 0.2% Triton X-100 in 1X PBS (PBST) in quick succession. Testes were fixed in freshly-made 4% paraformaldehyde (pH 7.4) in 0.5% Triton X-100 in 1X PBS for 30 minutes at room temperature. Successive washing in PBSTX (0.3% Triton X-100 + 0.1% Tween-20 in 1X PBS) and blocking steps were adopted from a previously detailed protocol^50^. Primary antibodies used in this study were rabbit anti-Vasa (1:2000, R. Lehmann), rabbit anti-CTPsyn (1:200, J.L. Liu), guinea pig anti-Traffic Jam (Tj) (1:5000, D. Godt), rabbit anti-Coilin (1:2000, J.G. Gall), guinea pig anti-Coilin (1:2000, J.G. Gall), mouse anti-Fasciclin III (1:40, Developmental Studies Hybridoma Bank (DSHB)), and mouse anti-1B1 (or hu-li tai shao (HTS)) (1:200, DSHB). Tissues were stained in primary antibody solutions overnight at room temperature. Secondary antibodies used were goat anti-rabbit DyLight 488, goat anti-rabbit DyLight 550 (1:600), goat anti-mouse DyLight 550 (1:600), goat anti-mouse DyLight 633, goat anti-guinea pig DyLight 488, and goat anti-guinea pig DyLight 633. All were purchased from Invitrogen and used at 1:400 dilutions, unless stated otherwise. After overnight nutation at room temperature, tissues were mounted with SlowFade Diamond Antifade Mountant (Thermo Fisher Scientific, USA, Cat. No. S36972).

### Microscopy

Images were acquired under 10X, 20X, 40X and 63X lenses on the LSM 710 confocal microscope platform (Zeiss, Oberkochen, Germany) with manufacturer-supplied software, Zen Blue (2012 Edition). Standard objectives were maintained, unless specified.

### Phenotyping assay

Males from crosses described above were dissected in batches of fifty. Testes were classified on the basis of apical tip size. A phenotypically significant testis was scored if the apical tip was bulging (or enlarged), i.e. the width or size of the apical tip of a testis was significantly larger than its testicular neck (or trunk). For instance, a light bulb-like shape (or phenotype) apical tip was considered a bulging testis. In the case where the tip was not bulged, an increase in size in the neck (immediately after the tip) was considered instead. This could closely resemble the cobra’s head with its neck expanded. Undeniably, both of the phenotypes mentioned would require the width to be significantly larger, easily observed under the dissecting microscope. Any other phenotypes that were found highly resemble a control testis, or show no significant increase in the size or width, was not considered a bulged testis.

### Quantifications of images and statistical analysis

Image processing and quantifications were carried out using Zen 2.3 SP1 (Black) and Zen Blue (2012) with its built-in tools. Statistical tests were conducted by presenting raw data into Excel sheets (Microsoft, CA). Two-way Student T-Tests was performed. Error bars represent the standard error of sum values. *p < 0.05 and **p < 0.01 were employed for significant differences.

### RNA extraction

Testes were dissected out into double-sterilized PBST within certain time constraints. A maximum of thirty males were processed per batch. PBST was replaced with ice-cold TRIzol® (Invitrogen, USA) and testes samples were immediately frozen at −20 °C. Three batches were consolidated prior to homogenization by QiaShredder spin columns. Subsequent RNA extraction was achieved using the RNeasy® Mini kit (all by Qiagen, Hilden, Germany). All extracts underwent DNase digestion (RNase-free DNase set, Qiagen Biotechnology, USA) prior to quantification.

### cDNA synthesis and qPCR

Complementary-DNA (cDNA) was synthesized with iScript™ Reverse Transcription Supermix (Bio-Rad, USA) according to manufacturer’s instructions. Primers utilized in quantitative-PCR (qPCR) are listed in Supplementary Table S1. Reactions were prepared in 10 µl volumes with iTaq™ Universal SYBR® Green Supermix before being loaded onto the CFX96 qPCR platform (all from Bio-Rad, Hercules, California, USA). Standard qPCR protocol for all plates entailed an initial denaturation of 2 minutes at 95 °C, followed by 40 cycles of fluorescence-quantification with denaturation, annealing and extension at 95 °C/10 s, 59 °C/15 s and 72 °C/15 s, respectively. Melting-curve analysis with temperatures between 65 °C to 95 °C succeeded every run. Data analysis was performed manually on Excel (Microsoft, California, USA) with aid from Bio-Rad’s own software where possible.

## Supplementary information

Supplementary Info
