# Supplementary material for: Ectopic miR-975 induces CTP synthase directed cell proliferation and differentiation in Drosophila melanogaster
Source: Sci Rep. 2019 Apr 15;9:6096. doi: 10.1038/s41598-019-42369-6 (PMC6465261; doi:10.1038/s41598-019-42369-6)
Supplement: Supplementary file 1 — Supplementary Info [file 41598_2019_42369_MOESM1_ESM.pdf]

Supplementary Information

Ectopic miR-975 induces CTP synthase directed cell proliferation and differentiation in *Drosophila melanogaster*.

Woo Wai Kan[1¥], Najat Dzaki[1¥], Shallinie Thangadurai[1] and Ghows Azzam[1*]

[1]*School of Biological Sciences, Universiti Sains Malaysia, 11800 Penang, Malaysia*

[¥]Equal contribution

*Corresponding author

Email: ghows@usm.my (G.A)

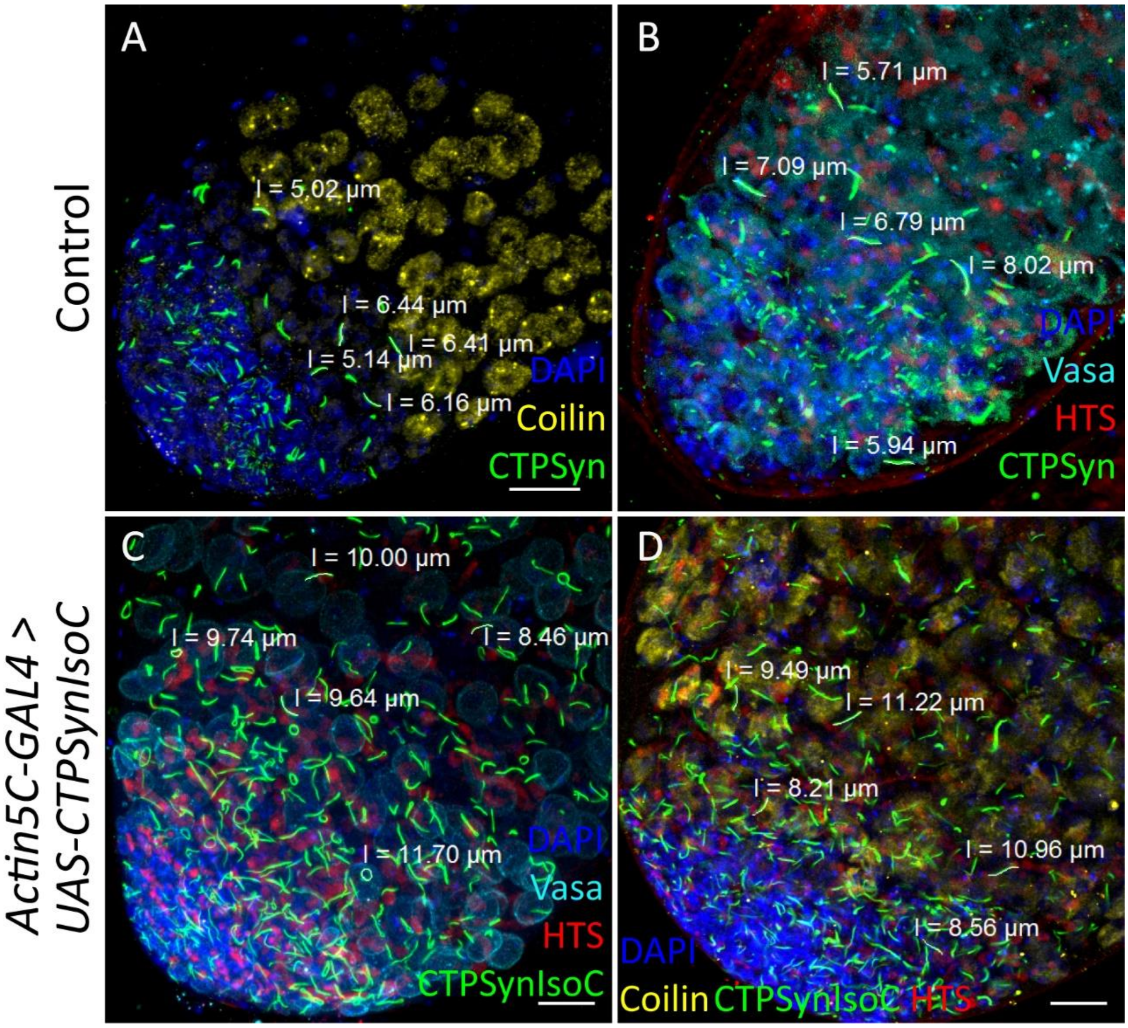

Supplementary Figure 1: Cytoophidia length in both control and transgenic testes. Random cytoophidia quantifications were not performed, rather the longest possible cytoophidia were picked. Lowercase letter L indicates length. Quantifications were were carried out using Zen 2.3 SP1 (Black).

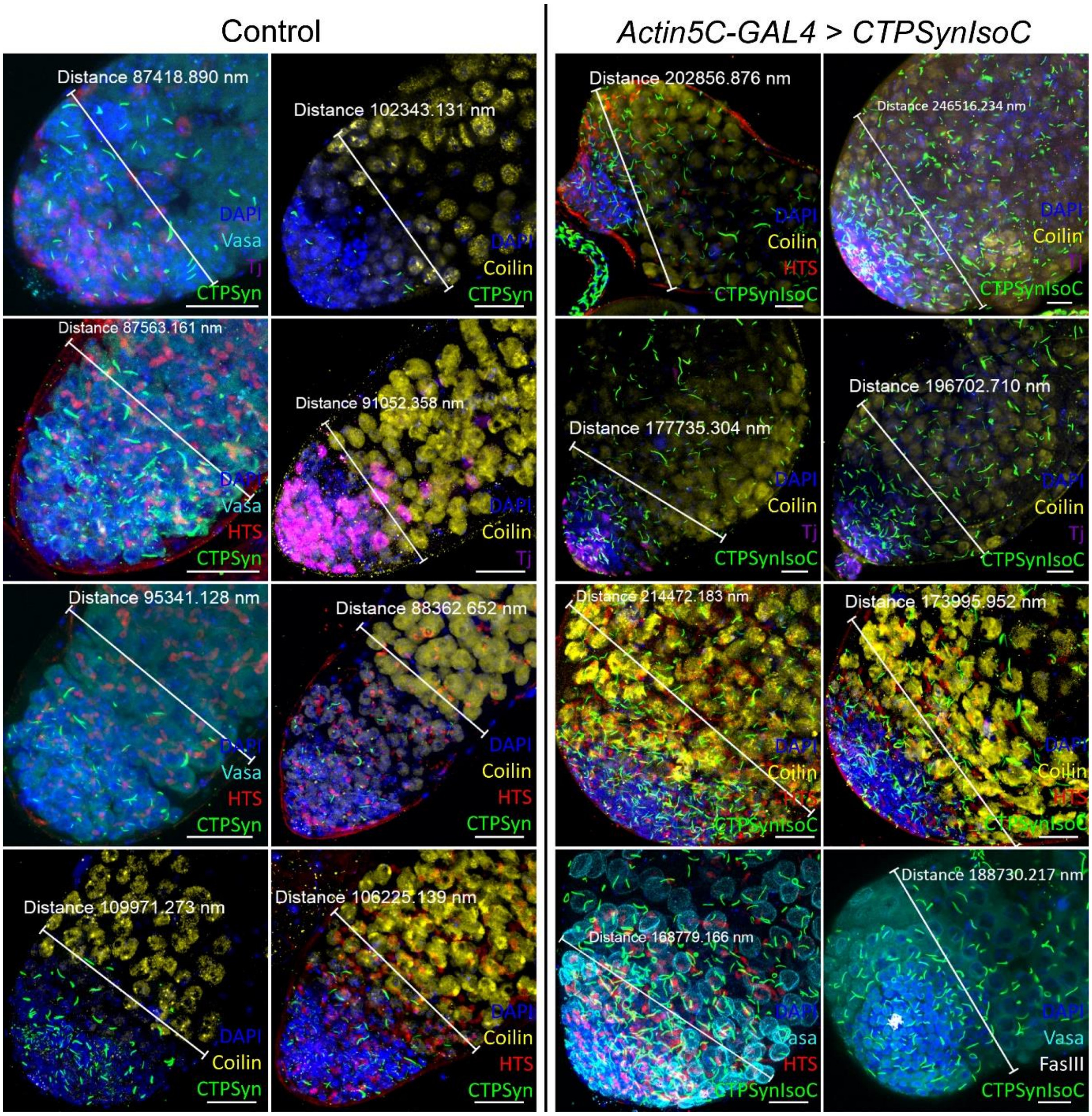

Supplementary Figure 2: Quantifications of testes diameter. A line was drawn across testis and DAPI channel was used as a reference. Quantifications were performed using Zen Blue (2012). Distance were indicated in nm.

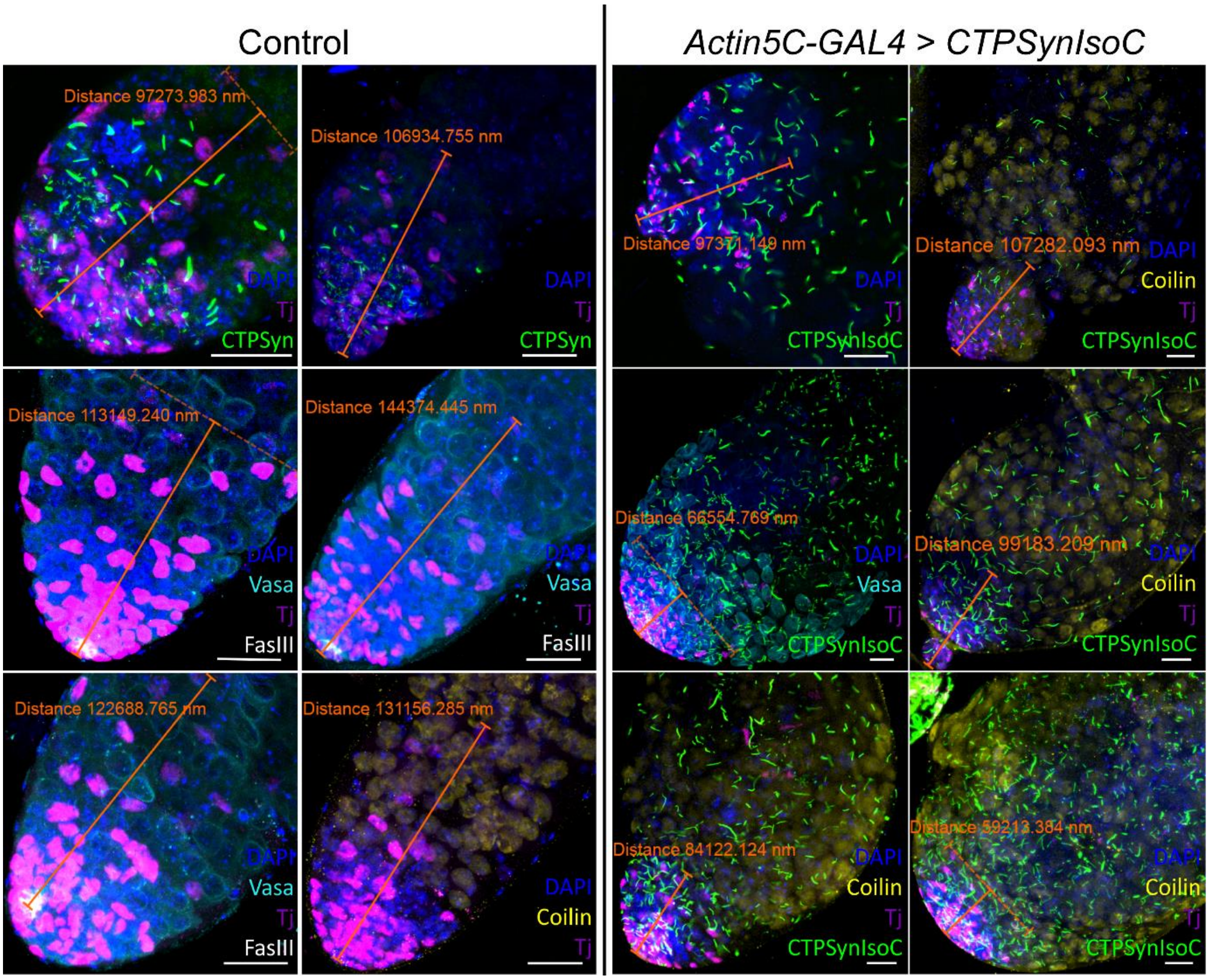

Supplementary Figure 3: Quantifications of Tj-positive cells spanned from apical tip. Distance spanned across the image to the last Tj-cells observed. Quantifications were performed using Zen Blue (2012). Distance were indicated in nm.

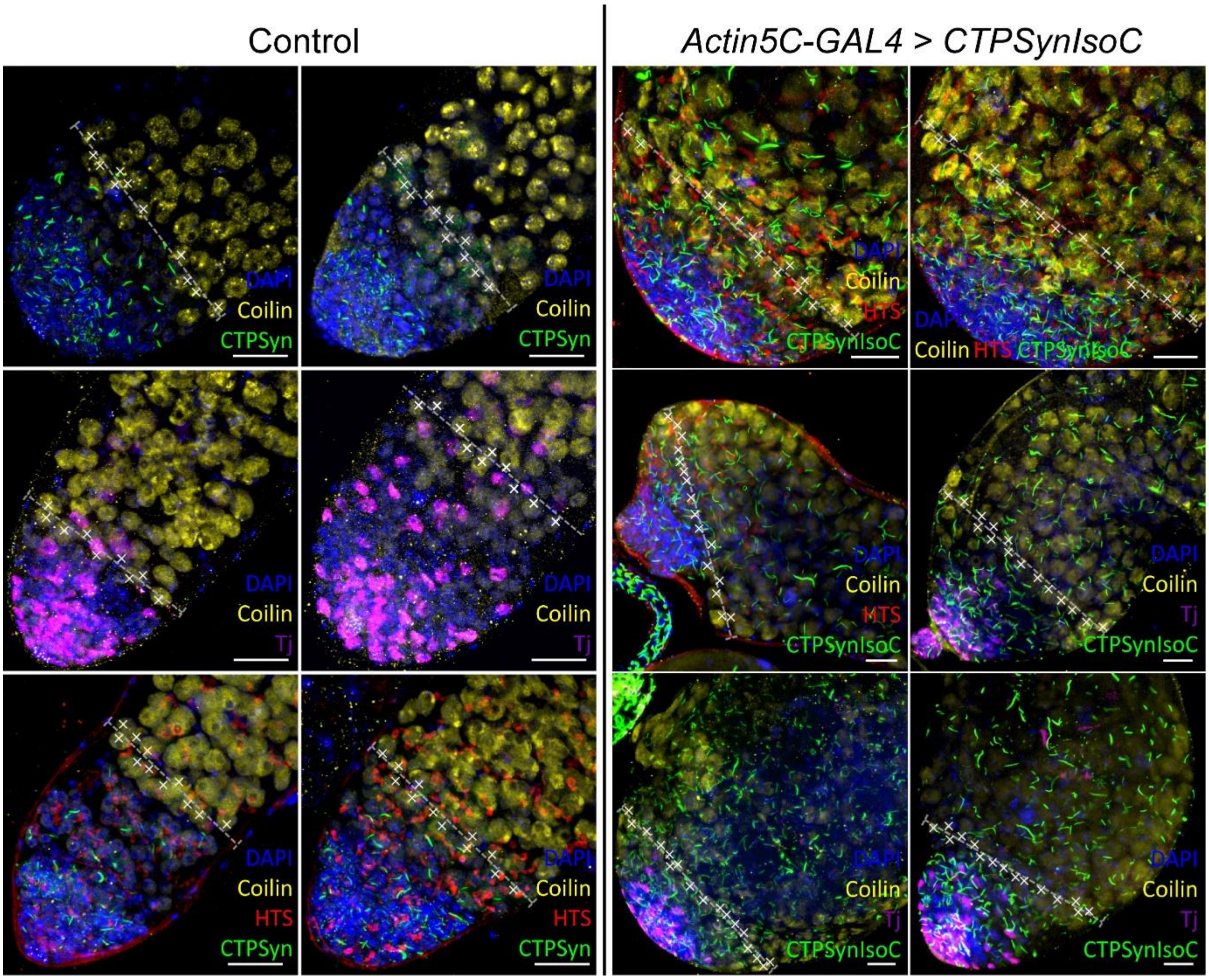

Supplementary Figure 4: Quantifications of Coilin-positive cells across the testicular width. A dashed line was drawn across the testis similar to diameter quantification, but anti-Coilin channels were used as a reference instead. A cell that was found to be on, or in contact with the dashed line was considered, and marked with "×".Quantifications were performed using Zen Blue (2012).

Supplementary Table S1: Primers for qPCR

Target	Primers (5' to 3')	Amplicon size (bp)
dAck (*Activated Cdc42 kinase*)	**FW: CCTCTGCATGCTCCTCTCTG**<br>**RV: TAGCCACTTGGATCCGTTCG**	166
Brat (*brain-tumour;* all isoforms)	**FW: CAACAAAGCAGTAAAGCTGTTAC**<br>**RV: CAGATTGCCGAATCGCTTGC**	172
IMPDH (*Inosine-5'-monophosphate dehydrogenase*)	**FW: ACGGTAACCGAATCGGAGATG**<br>**RV: CATGAAGCCGTGCTTGTACTT**	180
Myc (all isoforms)	**FW: TCAGATTCCGATGAGGAAATC**<br>**RV: GTGTACGGCAGATTGAAGTTAT**	176
AllCtps	**FW: AACGGTTCAAGTTGTCCCA**<br>**RV: GAACTGAAACTGACGGAAGG**	150
CTPsynIsoC	**FW: GAGTGATTGCCTCCTCGTTC**<br>**RV: TCCAAAAACCGTTCATAGTT**	156
Rp49 (*Ribosomal Protein 49*)	**FW: GCTAAGCTGTCGCACAAA**<br>**RV: GAACTTCTTGAATCCGGTG**	160
GAPDH *(Glyceraldehyde-3-phosphate dehydrogenase)*	**FW: AAGGGAATCCTGGGCTACAC**<br>**RV: ACCGAACTCGTTGTCGTACC**	147
